# Benign esophageal stricture model construction and mechanism exploration

**DOI:** 10.1038/s41598-023-38575-y

**Published:** 2023-07-20

**Authors:** Rui Wu, Min Fu, Hui-Min Tao, Tao Dong, Wen-Tao Fan, Li-Li Zhao, Zhi-Ning Fan, Li Liu

**Affiliations:** 1 Department of Digestive Endoscopy, The First Affiliated Hospital with Nanjing Medical University, Nanjing, 210029 Jiangsu China; 2 Department of Gastroenterology, The Fourth Affiliated Hospital of Nanjing Medical University, Nanjing, 210031 Jiangsu China; 3 Department of Critical Care Medicine, Jinling Hospital of Nanjing Medical University, Nanjing 210010 Jiangsu, China; 4 Department of Gynecology, The First Affiliated Hospital with Nanjing Medical University, Nanjing, 210029 Jiangsu China; 5 Digestive Endoscopy Center, Affiliated Hospital of Nanjing University of Chinese Medicine, Nanjing, 210004 Jiangsu China; 6 Department of General Surgery, The First Affiliated Hospital of Nanjing Medical University, Nanjing, 210029 Jiangsu China

**Keywords:** Gastrointestinal diseases, Gastrointestinal models

## Abstract

Esophageal stricture is a debilitating condition that negatively impacts patients' quality of life after undergoing endoscopic mucosal resection (EMR). Despite its significance, this disease remains underexplored due to the lack of a stable animal model. Under direct visualization with choledochoscopy, we retrogradely damaged the esophageal mucosal layer through the gastrostomy to create a rat model of esophageal stricture. The development of histological defects in the mucosal layer was assessed over a 2-week period after model induction. Then the models were evaluated using X-ray barium radiography, Hematoxylin–Eosin, Masson’s trichrome, Sirius red, and Victoria blue staining, multiphoton microscopic imaging. Additionally, the molecular mechanisms of esophageal stricture were explored by conducting RNA transcriptome sequencing, PCR, immunohistochemistry, and immunofluorescence staining. We successfully established fifteen rat models of esophageal stricture by injuring the mucosal layer. In the model group, the mucosal defect initially occurs and subsequently repaired. The epithelium was absent and was plastically remodeled by collagen during the acute inflammatory phase (Day 1), proliferation phase (Day 7), anaphase of proliferation (Day 10), and plastic remodeling phase (Day 14). We observed increased expression of COL1A1, acta2, FGF, IL-1, and TGF-β1 pathway in the model group. We established a highly repeatable rat model of esophageal stricture, and our results suggest that the mucosal defect of the esophagus is a critical factor in esophageal stricture development, rather than damage to the muscularis layer. We identified Atp4b, cyp1a2, and gstk1 as potential targets for treating esophageal stricture, while the TGF-β pathway was found to play an important role in its development.

## Introduction

Esophageal cancer ranks as the sixth leading cause of cancer-related mortality worldwide in 2020^1^. Although the patients with advanced esophageal cancer have a bleak prognosis, a 5-year survival rate of more than 90% can be expected for patients detected at an early stage^2^. With the advent of endoscopic operation^3^, the range of indications for endoscopic resection has gradually expanded as an alternative to surgery due to its minimally invasive nature^4^. However, the occurrence of esophageal strictures after endoscopic operation (due to the mucosal defect) significantly impacts patients' quality of life, causing dysphagia and necessitating multiple endoscopic dilations^5^.

Up to now, a reliable and readily available animal model of benign esophageal stricture has still been scarce, which makes it challenging to study the mechanisms and treatments for this condition. Currently, the major method for constructing an esophageal stricture model involves the creation of corrosive esophageal stricture by antegrade instillation^6^, which cannot be performed under direct visualization and is prone to significant heterogeneity. Additionally, aspiration and postoperative perforation are major limitations of this method based on our previous research experience. Some scholars have further used large animals such as pigs^7^ and dogs^8^ to construct esophageal stricture models, which are closer to clinical operations, but are limited in terms of sample size and difficult to scale up for exploratory research. In our study, we incorporated and expanded upon the previous methods for constructing esophageal stricture models and improved upon the research of Won et al.^9^ to establish a stable and reproducible rat model of esophageal stricture (we used an electrosurgical knife to remove the mucosal layer and submucosal layer of the esophagus, rather than induced necrosis of esophageal cells.), which has allowed us to analyze the molecular mechanisms involved in the formation of esophageal strictures.

## Methods and materials

The study was approved by the Ethics Committee of Nanjing medical University. All experiments were performed under the supervision of the Ethics Committee of Nanjing medical University in accordance with the relevant guidelines (IACUC-2103014, Institutional Animal Care and Use Committee of NMU). All methods are reported in accordance with ARRIVE guidelines. The rats were sacrificed with cervical dislocation in accordance with the relevant guidelines. Euthanasia was performed using pentobarbital sodium as the anesthetic agent. The rats were anesthetized with an intraperitoneal injection of 2% sodium pentobarbital at a dose of 0.2 ml/100 g.

### Constructing rat model

#### Preoperative preparation

Fifteen Sprague Dawley (SD) white male rats (8–10 weeks old, 250 ± 20 g) were obtained from Jiangsu Animal Experimental Center of Medical & Pharmaceutical Research (Nanjing, China). The rats were kept in the separate cages with controlled temperature (22 °C), 50% humidity, and a 12/12 h light/dark cycle. Prior to model construction, all rats underwent a 12-h fasting period.

#### Constructing rat model of esophageal stricture

**Figure 1:**
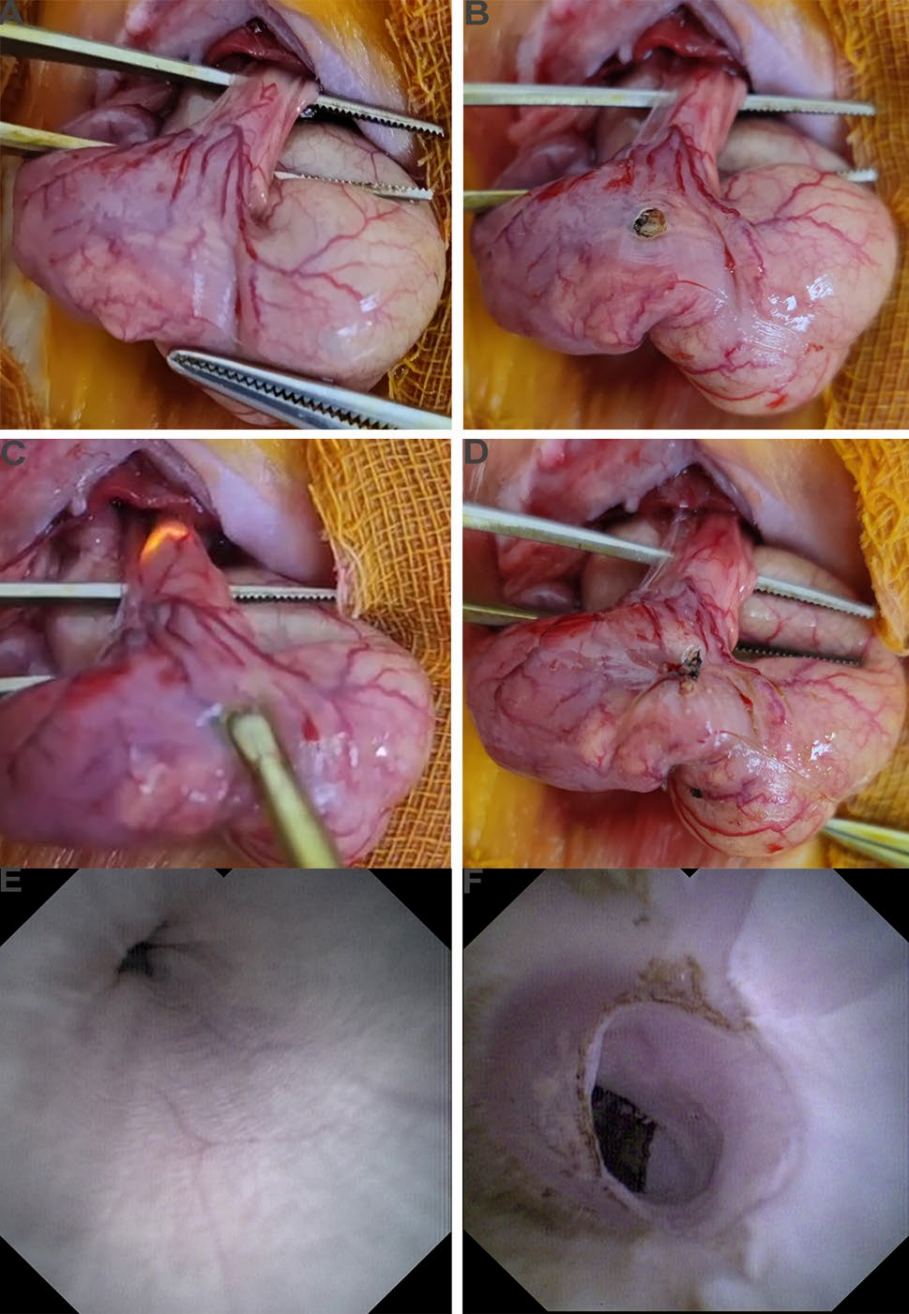
Constructing rat model of esophageal stricture. (**A**) The esophagus of rat was exposed. (**B**) The gastrostomy was performed. (**C**) The electric knife was performed for 0.5–1 s at the power of 150 W. (**D**) The gastrostomy fistula was sutured. (**E**) The normal esophagus under choledochoscope. (**F**) The esophageal mucosa was dissected after operation under choledochoscope.

The rats were anesthetized with an intraperitoneal injection of 2% sodium pentobarbital at a dose of 0.2 ml/100 g. The rats were placed in supine position and their abdomens were incised along the ventral midline. Gastrostomy was then performed using high frequency bipolar generator (LBS-G21-J(BK), Shanghai, China) (Supplement Fig. 1A). A flat electric knife (Supplement Fig. 1B) was inserted into the lower esophagus from the gastrostomy (approximately 4 cm), and maintained for 0.5–1 s at a power of 150 watts to produce peri-circumferential mucosal injury. The degree of injury was assessed by choledochoscope, and mucosal loss > 1/2 circumferential range was the primary criterion (Fig. 1E,F). Gastrostomy fistula and abdominal incision were then sutured and disinfected (Model Group). In the Sham Operation Group, rats underwent gastrostomy without esophageal mucosal injury. The rats were fasted for 72 h after operation, and were given 5% sugar saline containing fat milk, protein, and antibiotics (ceftiofur) in their drinking water. After completion of the fasting period, normal food was given, and the rats were weighed daily. X-ray barium radiography was performed 14 days after operation. Then, we longitudinally opened the esophagus and measured the circumference perpendicular to its longitudinal axis (i.e., perimeter). The criteria for success of esophageal stricture include: (1) the perimeter of stricture is less than half of the proximal esophagus in postmortem rats^10^; (2) pathological confirmation. Periscar tissue refers to the esophageal tissue located at least 3 cm away from the edge of the scar/stricture.

#### Constructing rat model of esophageal muscularis injury

The rats were incised along the ventral midline, and the esophageal muscularis were cut from the outside of the esophagus for 0.5–1 s at 150 W, with peri-circumferential muscularis injury (Esophageal Muscularis Injury Group). All other operations are the same as the construction of esophageal stricture by injuring the mucosa layer.

### Morphological and histological evaluation

The esophageal samples were fixed in 4% paraformaldehyde immediately after dissection of rats. Subsequently, the tissues were embedded in paraffin and sliced.

#### Hematoxylin–Eosin staining

The paraffin sections were dried (65 °C in the oven), deparaffinized in xylene, and rehydrated in the gradient alcohol (100–95–85–75%). Samples were stained with hematoxylin for 120 s, and rinsed with hydrochloric acid, 75% alcohol, and tap water for 10 s each. Then, the samples were stained with eosin for 5 min, placed in the gradient alcohol (85–95–100%) and xylene for dehydration.

#### Masson’s trichrome staining

Tissue specimens were dehydrated and stained according to the protocol and instructions of the Masson staining kit (JSENB, Hong Kong, China).

#### Sirius red staining

After the paraffin sections were deparaffinized and rehydrated, tissue specimens were stained with the Sirius red staining kit (Abcam, Shanghai, China).

#### Victoria blue staining

Tissue specimens were dehydrated and stained according to the protocol and instructions of the Victoria blue staining kit (BASO, Zhuhai, China).

The pathology slices were photographed by an electron microscope and analyzed by Image J software.

### The multiphoton microscopic imaging

The slices without staining were obtained from the paraffin of rat esophagus, and were prepared for multiphoton microscopic imaging^11^. The multiphoton microscopic system (Zeiss LSM 710 with upright Axio 200, Germany) was employed to image collagen and fluorescence components, separately. The channel with the wavelength range of 393–414 nm was used to presented the microstructure of collagen (green color coded), and another channel was used to exhibited the morphology of tissue components (the wavelength range of 438–708 nm, red color coded). 12-bit pixel depth and 2.56 μs per pixel were set. The fast fourier transform analysis was employed to reveal the collagen content of the esophageal stricture model.

### Immunohistochemistry staining

TGF-β1, α-sma, and COL1A1 immunohistochemistry (IHC) staining were performed after the samples were embedded in paraffin and sliced. Sections were placed in xylene for 3 times of 15 min each, absolute ethanol for 3 times, and subsequently rinsed with deionized water. The samples underwent antigen retrieval, and then were placed in 3% methanol hydrogen peroxide for 25 min, rinsed with deionized water, and placed in phosphate buffered saline (PBS) for 3 times. The slides were incubated with rabbit TGF-β1 (1:500 dilution, Servicebio, GB111876), α-sma (1:5000 dilution, Servicebio, GB111364), or COL1A1 (1:5000 dilution, Servicebio, GB11022-3) antibody at 4 °C overnight. The slides were washed in PBS for 3 times and incubated with the secondary antisera (1:200 dilution, Servicebio, GB23303) for 50 min at 37 °C. The slides were washed in PBS for 3 times, 3,3′-diaminobenzidine tetrahydrochloride (DAB) was applied, and rinsed in PBS and deionized water. The slides were stained with hematoxylin for 3 min as a counterstain, and washed in tap water. The pathology slices were photographed by an electron microscope and analyzed by Image J software.

### Immunofluorescence staining

The sections were prepared from embedded paraffin for immunofluorescence staining. After antigen retrieval and blocking, the sections were incubated with primary rabbit α-sma (1:5000 dilution, Servicebio, GB111364) or COL1A1 (1:5000 dilution, Servicebio, GB11022-3) antibody at 4 °C overnight, followed by incubation with the corresponding secondary antisera (1:200 dilution, Servicebio, GB23303) for 50 min at 37 °C. DAPI was then used to stain cell nuclei, and slices were photographed by confocal laser scanning fluorescence microscope.

### Polymerase chain reaction and RNA transcriptome sequencing

TRIzol method was used to extract RNA of six rat models, which then was subjected to reverse transcription. QRT-PCR was performed using SYBR Green in the QuantStudio (TM) 7 Flex System following manufacturer’s protocols and comparative Cт (ΔΔCт) was used to analyze the results. Internal control was β-actin. Furthermore, transcriptome sequencing was performed. This study was the first to perform a transcriptome analysis of esophageal stricture, which identified 2628 differentially expressed genes (DEGs) in esophageal stricture tissues compared with periscar tissues in the model group (n = 3). The standard of |log2 fold change (logFC)|≥ 1 and adjusted P value ≤ 0.05 was set for significant DEGs according to the normalized gene expression levels.

### Protein–protein interaction (PPI) network construction

PPI was performed in the top 200 DEGs using the String database (https://string-db.org/). The hub 100 genes were identified based on the degree, and the PPI network was illustrated and visualized by Cytoscape software (version 3.5.0).

### Statistics

The statistical analysis was performed using SPSS software 25.0 version (IBM Corp, Armonk, NY, USA). Categorical variables were analyzed using chi-square test or Fisher’s exact test, and continuous variables were analyzed using t-test. The level of statistical significance was set at P < 0.05.

### Ethical approval

The study was approved by the Ethics Committee of Nanjing medical University. All experiments were performed under the supervision of the Ethics Committee of Nanjing medical University in accordance with the relevant guidelines (IACUC-2103014, Institutional Animal Care and Use Committee of NMU). All methods are reported in accordance with ARRIVE guidelines. The rats were sacrificed with cervical dislocation in accordance with the relevant guidelines. Euthanasia was performed using pentobarbital sodium as the anesthetic agent. The rats were anesthetized with an intraperitoneal injection of 2% sodium pentobarbital at a dose of 0.2 ml/100 g.

## Results

### Constructing rat model of esophageal stricture

Fifteen rat models were established by injuring mucosal layer. First, a midline abdominal incision was made to expose the esophagus and stomach (Fig. 1A). Then, a gastrostomy was performed (Fig. 1B). Under direct visualization with choledochoscopy (Fig. 1E,F), we retrogradely damaged the esophageal mucosal layer (Fig. 1C). Finally, the incision was sutured (Fig. 1D). According to the pathology, the mucosal layer of the esophagus was removed, which is consistent with EMR operation (Supplement Fig. 1C,D).

### Pathological progress of esophageal stricture in rats

**Figure 2:**
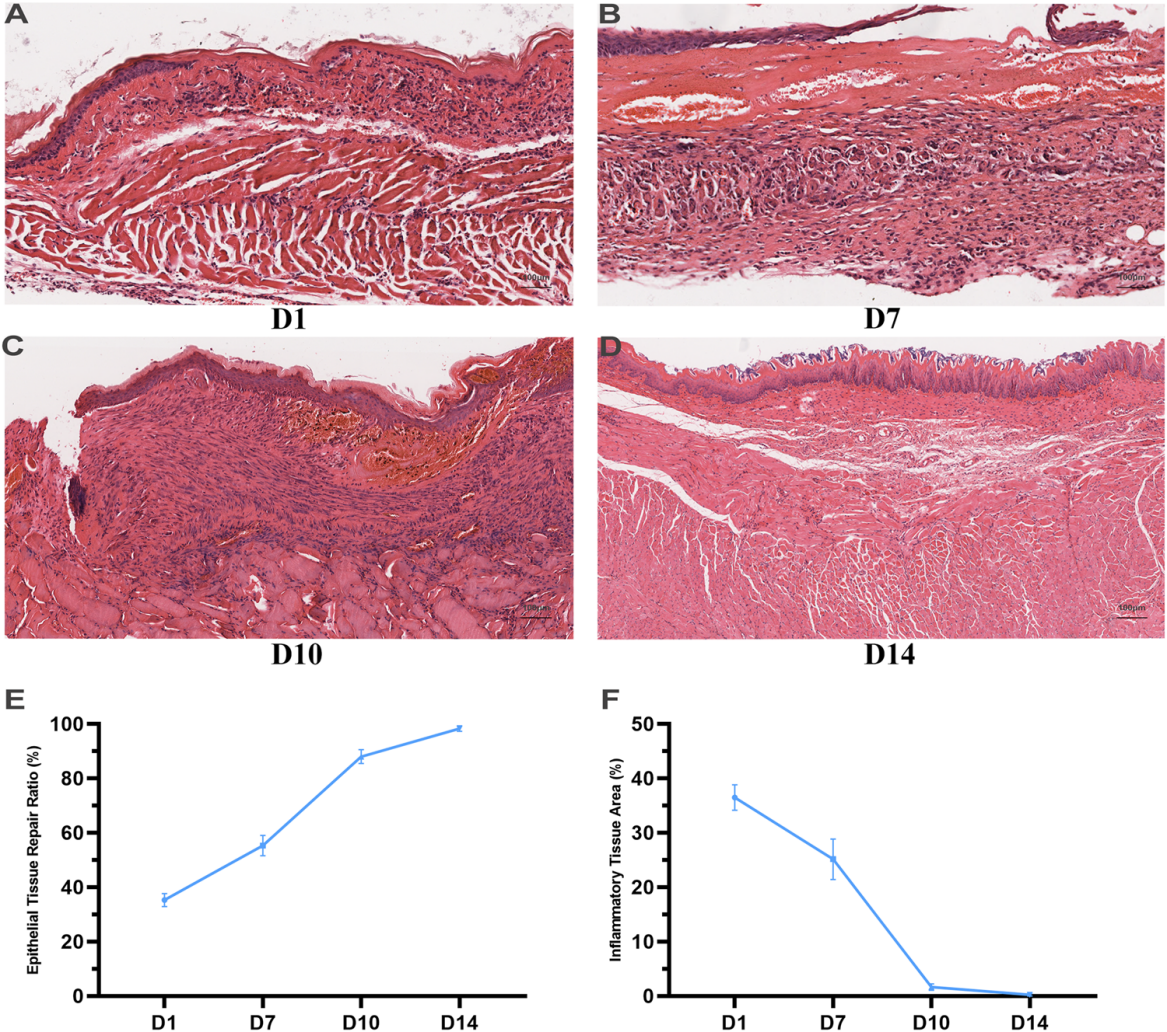
Pathological progress of esophageal stricture in rats. (**A**) The rats in the acute inflammatory phase (Day 1). Regenerated epithelium was disrupted, and the hyperemia and edema occurred in submucosa layer. (**B**) The rats in the proliferation phase (Day 7 in model group). The extensive granulation tissue formation. (**C**) The rats in the anaphase of proliferation (Day 10 in model group). Fibroblasts proliferated, new capillaries increased, and regenerated epithelium was repaired. (**D**) The rats in the plastic remodeling phase (Day 14 in model group). Abundant fibrous connective tissue appeared. (**E**) The epithelial layer underwent gradual repair. (**F**) The inflammatory area gradually decreased.

Rats in model group (n = 12) and sham operation group (n = 12) were killed and dissected on the first, seventh, tenth, and fourteenth day after operation (3 rats each time). In the acute inflammatory phase (Day 1), regenerated epithelium was disrupted, and the defected region of mucosal layer was observed. The hyperemia and edema occurred in submucosa layer, which was infiltrated by inflammatory cells (Fig. 2A). In the proliferation phase (Day 7), extensive granulation tissue formation and partial fibrosis were observed in the margin of defected region in mucosal layer and submucosa layer, and new capillaries increased. In additional, inflammatory cell infiltration decreased (Fig. 2B). In anaphase of proliferation (Day 10), fibroblasts proliferated, new capillaries increased, and regenerated epithelium was repaired (Fig. 2C). In the plastic remodeling phase (Day 14), abundant fibrous connective tissue appeared, and inflammatory cells and new capillaries significantly reduced (Fig. 2D). After the induction of esophageal stricture, a discontinuity in the mucosal layer of the esophagus was observed (Supplement Fig. 1C,D). Subsequently, the epithelial layer underwent gradual repair, leading to complete restoration, while the inflammatory area gradually decreased (Fig. 2E,F).

### General morphology of model rats

**Figure 3:**
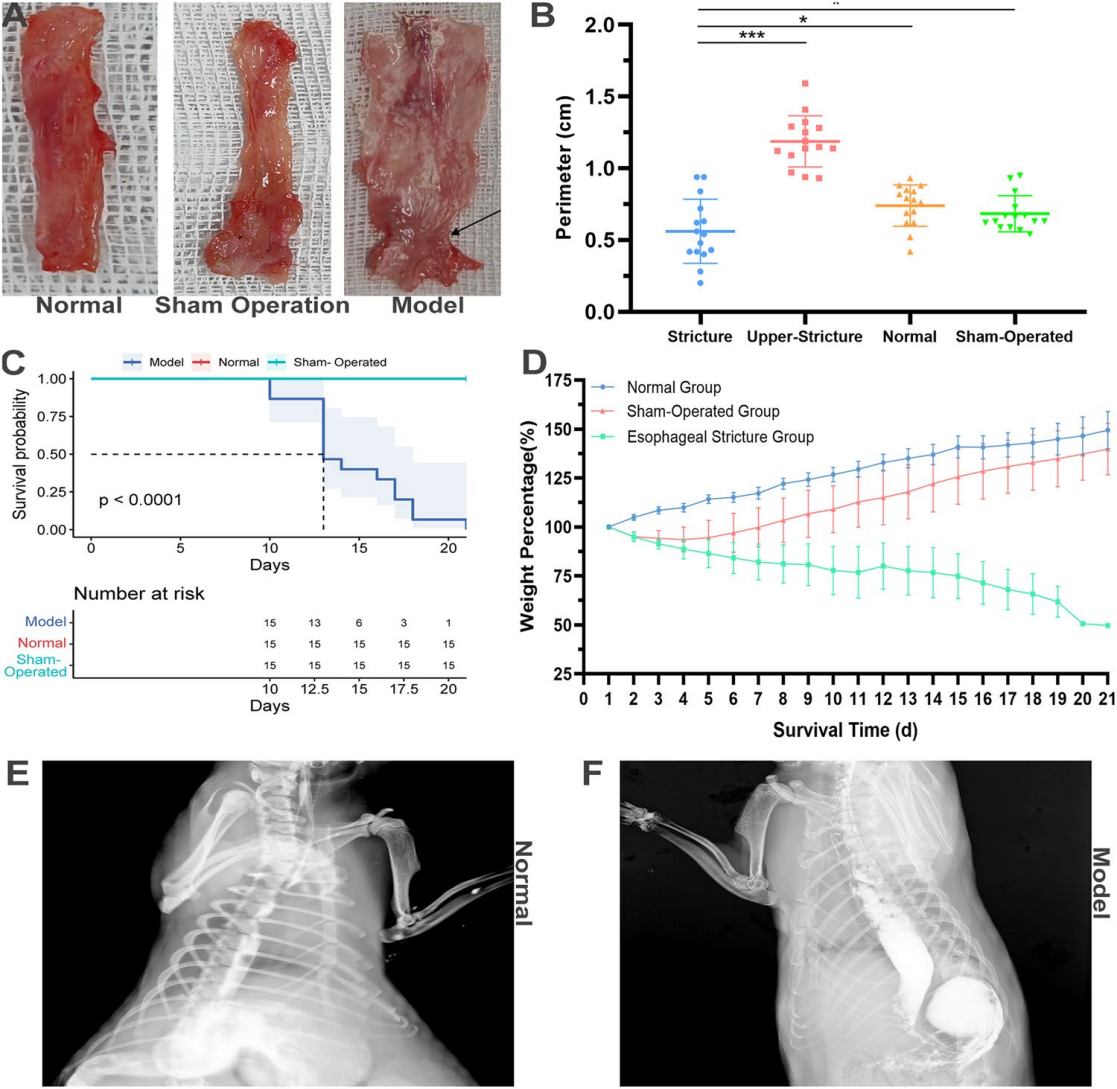
General morphology of model rats. (**A**) The general morphology of esophagus in normal, sham operation, and model groups. The constriction ring was pointed out by the arrow. (**B**) The perimeter of esophagus in normal, sham operation, and model groups. (**C**) The KM plotter of rats in normal, sham operation, and model groups. (**D**) The change of body weight in normal, sham operation, and model groups in 21 days after operation. (**E**) X-ray barium radiography in normal group. (**F**) X-ray barium radiography in model group.

No significant constriction ring was observed in either the normal or sham groups (Fig. 3A), however, in the model group, the constriction rings were observed in the abdominal esophagus, and the cervical and thoracic esophagus were dilated with residual water and food based on the dissection (Fig. 3A). The perimeter of the constriction rings was less than the upper esophagus in model group, and it was also less than the abdominal esophagus in the normal group and the sham operation group (Fig. 3B). The length of constriction rings was 0.34 ± 0.15 cm. The survival time of rats was 14.60 ± 3.11 day (range 10–21 days) (Fig. 3C), and their weight was gradually lost over 21 days (Fig. 3D). X-ray barium radiography showed that the esophageal stricture in the model rats, while the esophagus of the normal rats was smooth without dilation (Fig. 3E,F).

### Pathological features of rats with esophageal stricture

**Figure 4:**
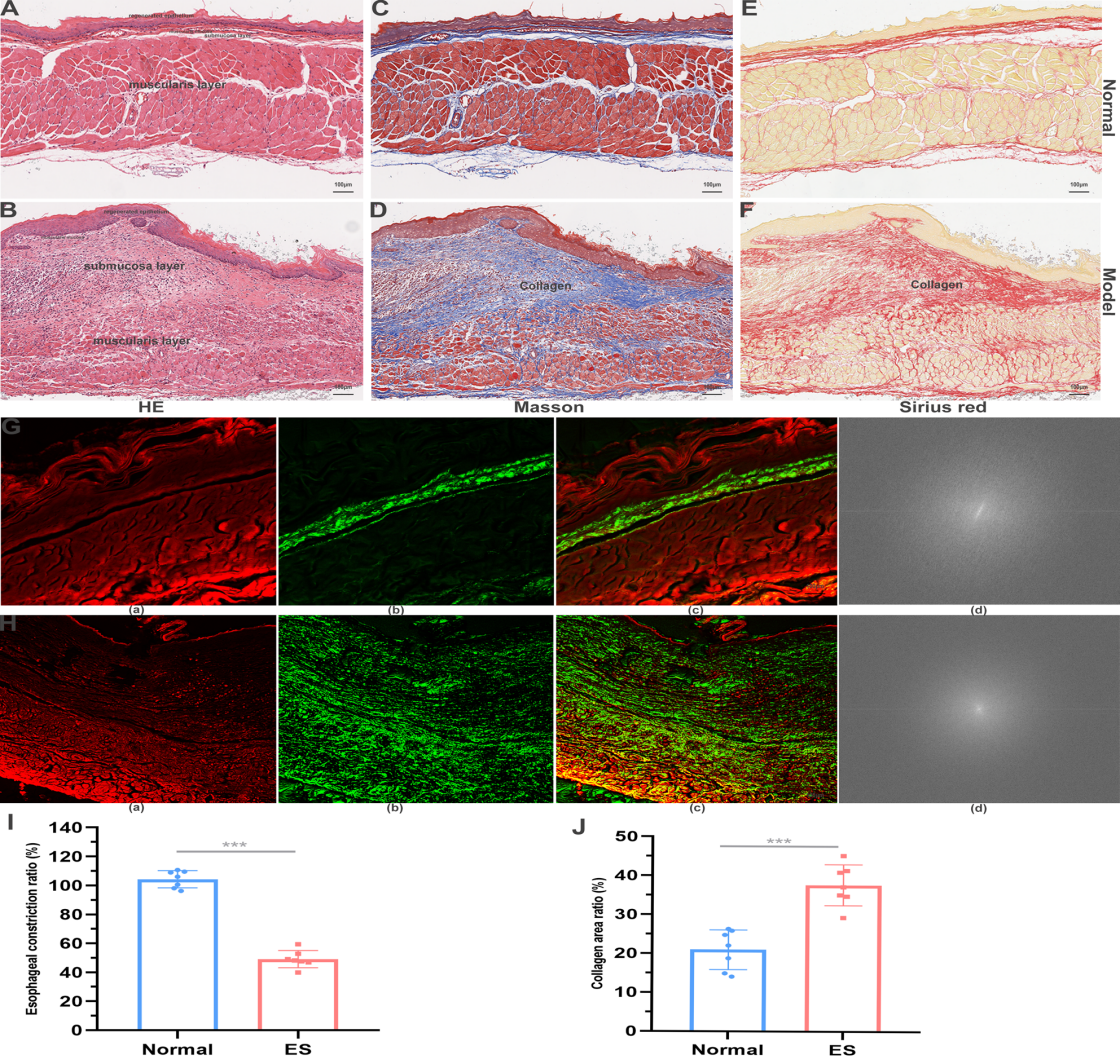
The collagen of esophagus increases in the scar tissue in the model group. (**A**) The HE of periscar tissue for rats in model group. (**B**) The HE of scar tissue for rats in model group. (**C**) The Masson’s trichrome staining of periscar tissue for rats in model group. (**D**) The Masson’s trichrome staining of scar tissue for rats in model group. (**E**) The Sirius red staining of periscar tissue for rats in model group. (**F**) The Sirius red staining of scar tissue for rats in model group. (**G**,**H**) The multiphoton microscopic imaging of periscar and scar tissue for rats in model group. (**a**) The morphology of tissue components (red color coded). (**b**) The microstructure of collagen (green color coded). (**c**) The merged image of (**a**,**b**). (**d**) The fast fourier transform analysis of collagen. (**I**) The esophageal constriction ratio was decreased in the model group. (**J**) the collagen area was increased significantly in the model group.

In the scar of esophageal stricture, epithelium was disrupted and injured, and then plastically remodeled by collagen. The muscularis mucosae layer was disrupted, and the fibrous tissue was throughout the layer beneath the regenerated epithelium. In the muscularis propria layer, inflammatory cell infiltration and fibrosis were not found. Furthermore, the submucosal layer shows obvious thickening and structural disorder. However, the inflammation has subsided and scar tissue is visible. There is an increase in fiber bundles in the muscle layer, but no other significant abnormalities were observed (Fig. 4A,B). According to the Masson’s trichrome staining, Sirius red staining, and Victoria blue staining, the collagen was increased in the scar of esophageal stricture compared with the periscar tissue (Fig. 4C–F; Supplement Fig. 2). The esophageal constriction ratio was decreased, while the collagen area was increased significantly in the model group (Fig. 4I,J). In addition, multiphoton microscopic imaging also confirmed that the collagen was increased in the scar of model rats, and its collagen structure is different from the collagen in the normal esophagus (Fig. 4G,H).

### Fibrosis related genes and pathways were significantly up-regulated in esophageal stricture models

**Figure 5:**
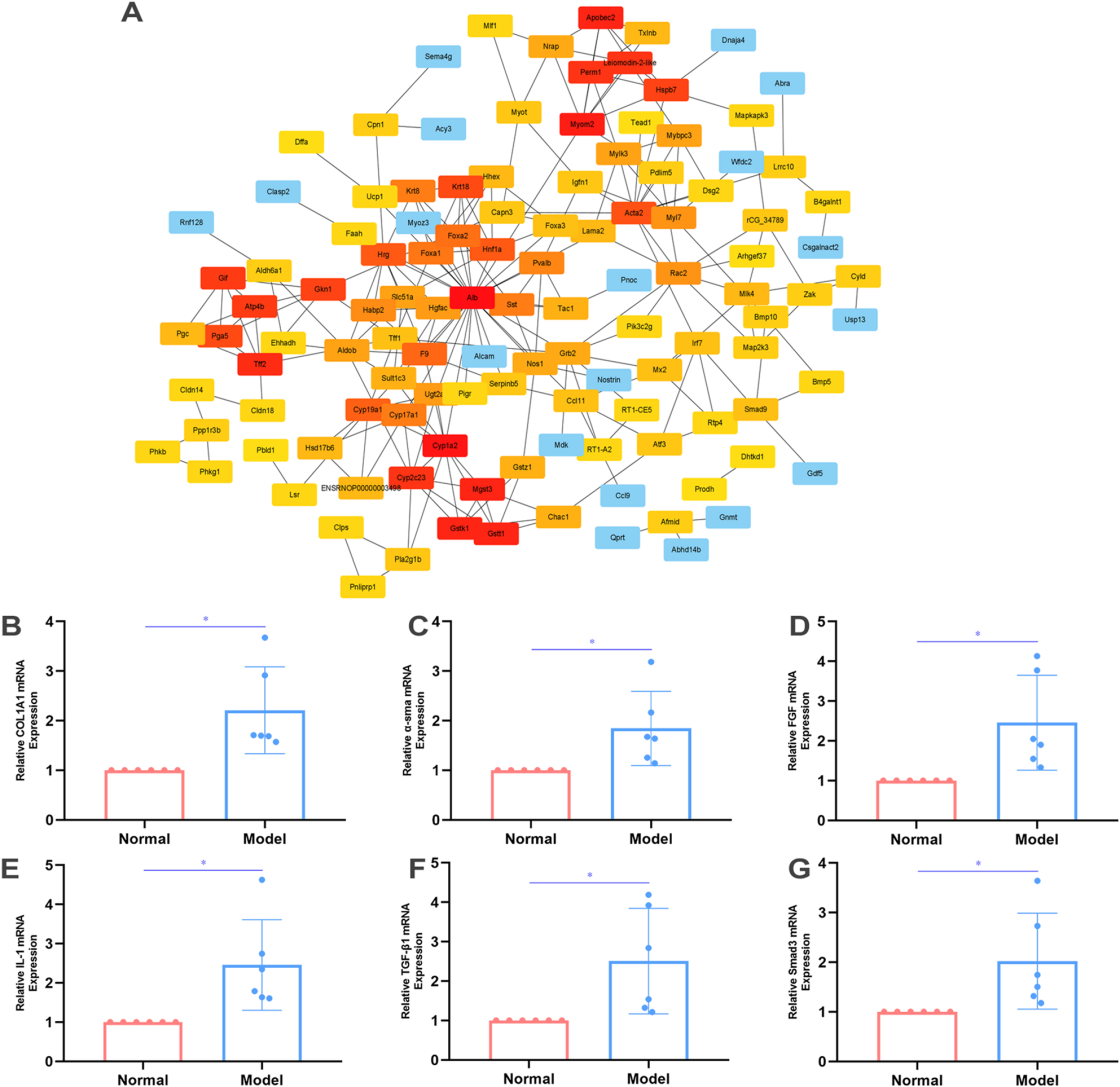
Transcriptome analysis of esophageal stricture. (**A**) The PPI network was conducted using the top 200 DEGs, and the hub 100 genes were explored. (**B**–**G**) The qRT-PCR were performed in the scar tissue and periscar tissue in the model group (n = 6), including COL1A1, α-sma, fibroblast growth factor (FGF), IL-1, TGF-β1, and smad3, respectively.

To the best of our knowledge, this study was the first to perform a transcriptome analysis of esophageal stricture, which identified 2628 differentially expressed genes (DEGs) in esophageal stricture tissues compared with periscar tissues in the model group. Gene ontology (GO) analysis was performed in DEGs, which revealed enrichment of myofibril (GO:0030016, rich factor = 0.433), indicating that our model accurately reflected the main characteristics of esophageal stricture. The top 200 DEGs were used to construct a PPI network, and the hub genes were identified based on the top 100 DEGs (Fig. 5A). The genes of acta2, myom2, mylk3, mybpc3, and myl7 were defined as the hub genes, which were associated with fibrosis. Additionally, bmp and smad were also identified as hub genes that were linked to the TGF-β pathway. Notably, atp4b, cyp1a2, and gstk1 were found to be potential targets for the treatment of esophageal stricture.

In the scar of esophageal stricture, the expression of genes associated with fibrosis and inflammation was found to be increased compared with that in periscar tissue. Myofibroblast contributes to altered microarchitecture of collagen type I^12^, and excessive synthesis, deposition, and remodeling of extracellular matrix proteins in fibrosis^13^. The acta2 is the biomarker for myofibroblasts. TGF-β signaling has been well studied in initiating and sustaining myofibroblast formation and function^14,15^, but its role in esophageal stricture has been poorly understood. In our study, PCR analysis showed that COL1A1, acta2, fibroblast growth factor (FGF), IL-1, TGF-β1, and smad3 were all increased (Fig. 5B–G).

**Figure 6:**
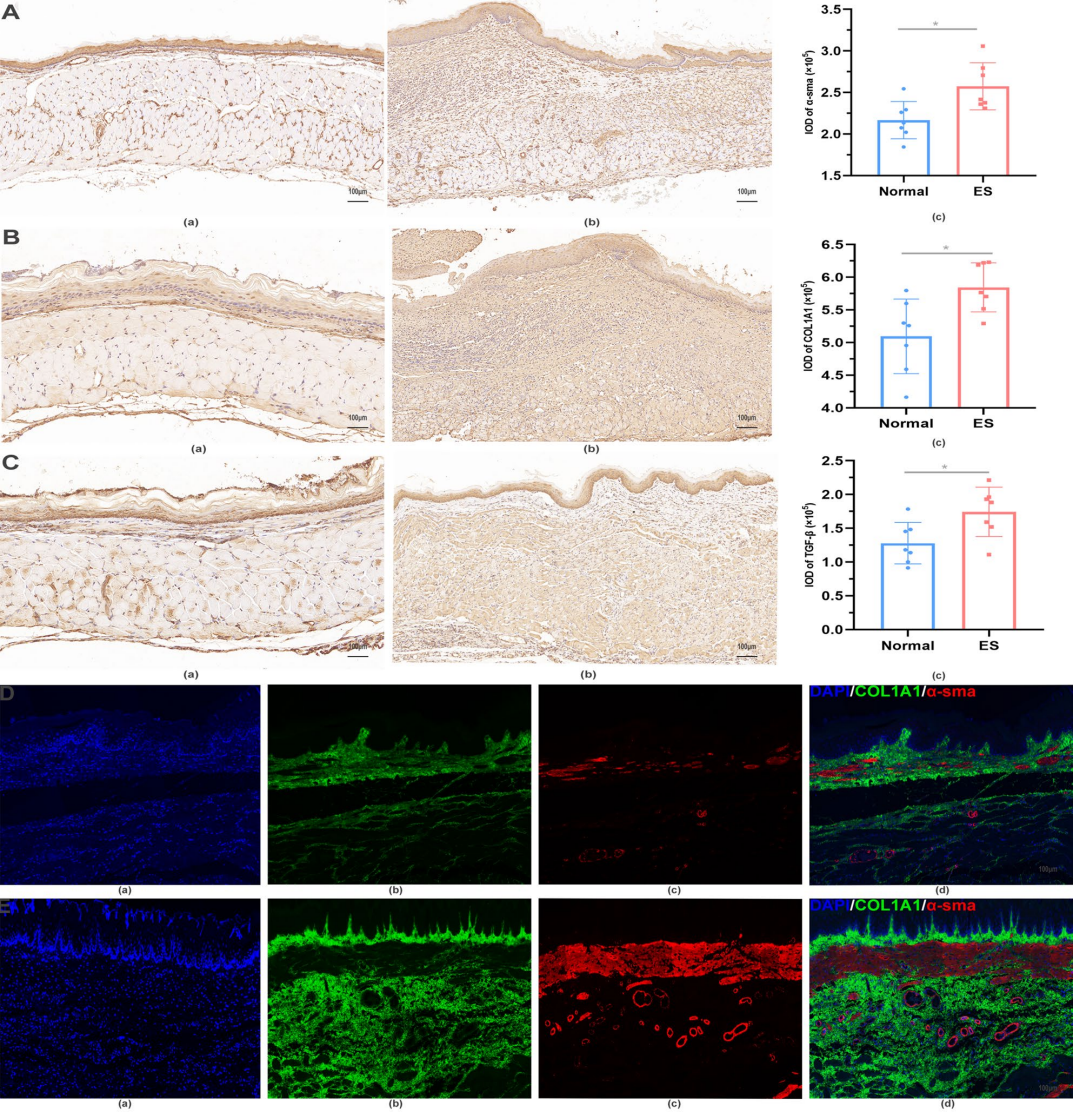
The immunohistochemistry and immunofluorescence staining of rats in the normal group and model group. (**A**) The α-sma of rats. (**a**) The normal group. (**b**) The model group. (**c**) IOD of α-sma. (**B**) The COL1A1 of rats. (**a**) The normal group. (**b**) The model group. (**c**) IOD of COL1A1. **C**) The TGF-β1 of rats. (**a**) The normal group. (**b**) The model group. (**c**) IOD of TGF-β1. (**D**,**E**) The immunofluorescence staining of DAPI (**a**), COL1A1 (**b**), and α-sma (**c**) in the normal group and model group. The merged image of DAPI, COL1A1, and α-sma was shown in (**d**).

Moreover, IHC and immunofluorescence staining revealed increased levels of acta2 and COL1A1 in the scar of esophageal stricture, indicating that myofibroblasts and collagen were both increased in this tissue (Fig. 6A,B; D, E). In addition, TGF-β1 was found to be significantly increased in esophageal stricture tissue compared with periscar tissue (Fig. 6C).

### The esophageal muscularis injury model

**Figure 7:**
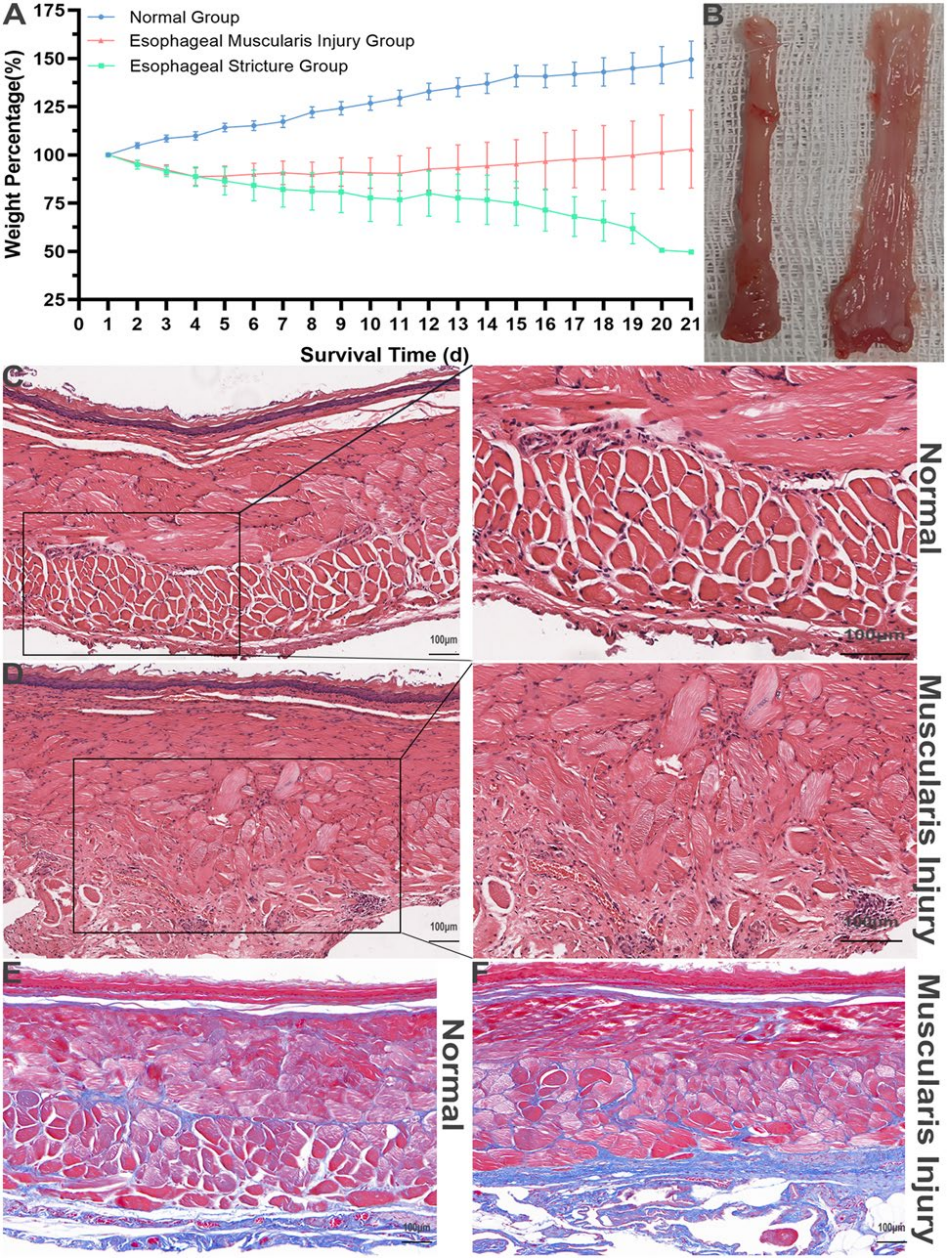
The esophageal muscularis injury model. (**A**) The change of body weight in normal, muscularis injury, and model groups in 21 days after operation. (**B**) The general morphology of rats with esophageal muscularis injury (Day 21). (**C**) The HE of normal rats (Day 21). (**D**) The HE of esophageal muscularis injury rats (Day 21). (**E**) The Masson’s trichrome normal rats (Day 21). (**F**) The Masson’s trichrome staining of esophageal muscularis injury rats (Day 21).

Interestingly, our study showed that when the esophageal muscularis was injured, esophageal stricture could not be induced (Fig. 7A,B). 10 rats were constructed the esophageal muscularis injury model. Their body weight was decreased during the first 4 days as the rats in the model group, and then followed by gradual increase (Fig. 7A). However, on the 21st day post-surgery, none of the rats showed any signs of esophageal stricture (Fig. 7B). According to postoperative pathology, there was no obvious collagen hyperplasia or remodeling in Day 21 (Fig. 7E,F), although there was obvious muscularis disorder (Fig. 7C,D).

## Discussion

Esophageal stricture was induced by fibrotic changes and scar formation, which starting with inflammation, followed by proliferation and remodeling^16^. In addition, biomechanical deterioration and the change of collagen components in the submucosal layer also result in the pathogenesis of esophageal stricture^17^. However, the molecular mechanism of esophageal stricture remains underexplored due to the lack of a stable animal model.

In previous studies, it has been difficult to conduct large-sample research on esophageal stricture in large animals due to high costs and difficulty in acquisition. Currently, the most dominant method for esophageal stricture modeling is the use of sodium hydroxide corrosive strictures^10,18^. However, according to our study, this method was limited due to the lack of direct observation during the operation and the individual differences in rat esophagus, which resulted in high mortality and heterogeneity (Supplement Fig. 3A). In addition, it was difficult to detect the progression of esophageal stricture in a simple and effective manner after the operation, which limits the applicability of this method. Furthermore, simulating clinical EMR procedures in small animals such as rats results in significant trauma, which is difficult to repeat and thus not suitable as the primary method for esophageal stricture modeling (Supplement Fig. 3B).

We incorporated and expanded upon the previous methods for constructing esophageal stricture models and improved upon the research of Won et al.^9^ to establish a stable and reproducible rat model of esophageal stricture (i.e., retrograde esophageal stricture modeling approach). This method enables the assessment of esophageal damage during the operation under direct vision, and the degree of esophageal mucosal damage can be observed through choledochoscope, which addresses the heterogeneity in esophageal stricture modeling caused by individual differences in rats. Additionally, the modeling process under observation greatly reduces the occurrence of postoperative complications such as esophageal fistula. Furthermore, the healing process of the abdominal incision roughly reflects the progression of esophageal stricture and provides a simple and convenient way to monitor the progression of esophageal strictures in vitro to some extent. This method has a significantly higher success rate (15/15, 100%) than corrosive strictures (3/15, 20%) and surgical methods (1/15, 6.7%) and is an effective modeling method for esophageal strictures (Supplement Fig. 3A,B, Fig. 1).

Furthermore, exploring the pathological progression of esophageal stricture is an urgent and essential component to fully comprehend and treat this disease, yet it has not been studied comprehensively to date. According to our study, the cuticle, mucosal, and submucosal layer of rats were absent (Fig. 1A, Supplement Figs. 1C,D, 2A), and the mechanism of stricture was similar to EMR operation^19,20^. Then, esophageal stricture underwent acute inflammatory phase (Day 1), proliferation phase (Day 7), anaphase of proliferation (Day 10), and plastic remodeling phase (Day 14). During the first phase, inflammatory cells infiltrated the affected area, while extensive granulation tissue was observed in the initial week. Subsequently, fibroblast proliferation occurred, leading to the eventual appearance of abundant fibrous connective tissue^21^.

In accordance with previous investigations^22,23^, a significant increase in submucosal collagen was observed in esophageal stricture (Fig. 4). Despite exhibiting elevated levels of collagen compared to a normal esophagus, esophageal scars are unable to achieve the same tensile strength as normal esophagus due to the tightly-parallel alignment of collagen bundles (i.e., “basketweave” orientation of collagen in unwounded tissue)^24,25^. Our study utilized multiphoton microscopic imaging, fast Fourier transform analysis, and differential staining techniques to determine that the collagen structure in model rats had been altered (Fig. 4). Additionally, RNA transcriptome sequencing was first performed in the rats with benign esophageal stricture. The genes and pathway associated with fibrosis were explored such as acta2 and TGF-β1 pathway^23^. According to previous reports, the differentiation of fibroblasts into acta2-expressing contractile-myofibroblasts plays a crucial role in tissue repair. However, uncontrolled expansion of myofibroblasts can lead to excessive tissue scarring and the development of esophageal stricture^26,27^. Furthermore, TGF-β1, Col1a1, and Acta2 were found to be increased in the tissue with esophageal stricture based on PCR and histological analysis. These findings indicate that our rat model successfully simulated benign esophageal stricture.

In addition, during the acute inflammatory phase, the regenerated epithelium was disrupted, and subsequently repaired during the subsequent phase, which is a key factor leading to esophageal stricture. Previous studies have indicated that a circumferential range > 3/4^28,29^ and submucosal infiltration^20,30^ are the main risk factors for esophageal stricture after endoscopic resection. Interestingly, our study showed that when the esophageal muscularis was injured, esophageal stricture could not be induced (Fig. 7). 10 rats were constructed the esophageal muscularis injury model. Their body weight was decreased during the first 4 days as the rats in the model group, and then followed by gradual increase (Fig. 7A). However, on the 21st day post-surgery, none of the rats showed any signs of esophageal stricture (Fig. 7B). According to postoperative pathology, there was no obvious collagen hyperplasia or remodeling in Day 21 (Fig. 7E,F), although there was obvious muscularis disorder (Fig. 7C,D). The incidence of esophageal stricture after transthoracic esophagectomy was reported to be low (20%^31^), which may be due to less mucosal layer loss. Liu et al.^17^ further reported that severe strictures would develop if the overlying mucosa of esophagus was resected, while no esophageal stricture would develop if the mucosa was incised but not resected. Our findings support this notion and suggest that the extent of loss of esophageal mucosa is a critical factor for esophageal stricture^32^, not just the damage of the esophageal mucosa (Fig. 1A, Supplement Fig. 1C,D, Fig. 2).

Our research presents a novel and consistent animal model of benign esophageal stricture in rats, which is stable and reproducible, with a success rate of more than 90%. Furthermore, this model effectively eliminates the risk of esophageal fistula formation, as well as the occurrence of modeling site heterogeneity. Importantly, our study provides a comprehensive exploration of the underlying mechanisms of esophageal stricture, offering valuable insights for the development of novel therapeutic approaches to manage this disease.

## Supplementary Information

Supplementary Figure 1.

Supplementary Figure 2.

Supplementary Figure 3.

Supplementary Legends.

## Data Availability

The datasets used and/or analysed during the current study available from the corresponding author on reasonable request.
