# Supplementary figures and images for: Benign esophageal stricture model construction and mechanism exploration
Source: Sci Rep. 2023 Jul 20;13:11769. doi: 10.1038/s41598-023-38575-y (PMC10359281; doi:10.1038/s41598-023-38575-y)

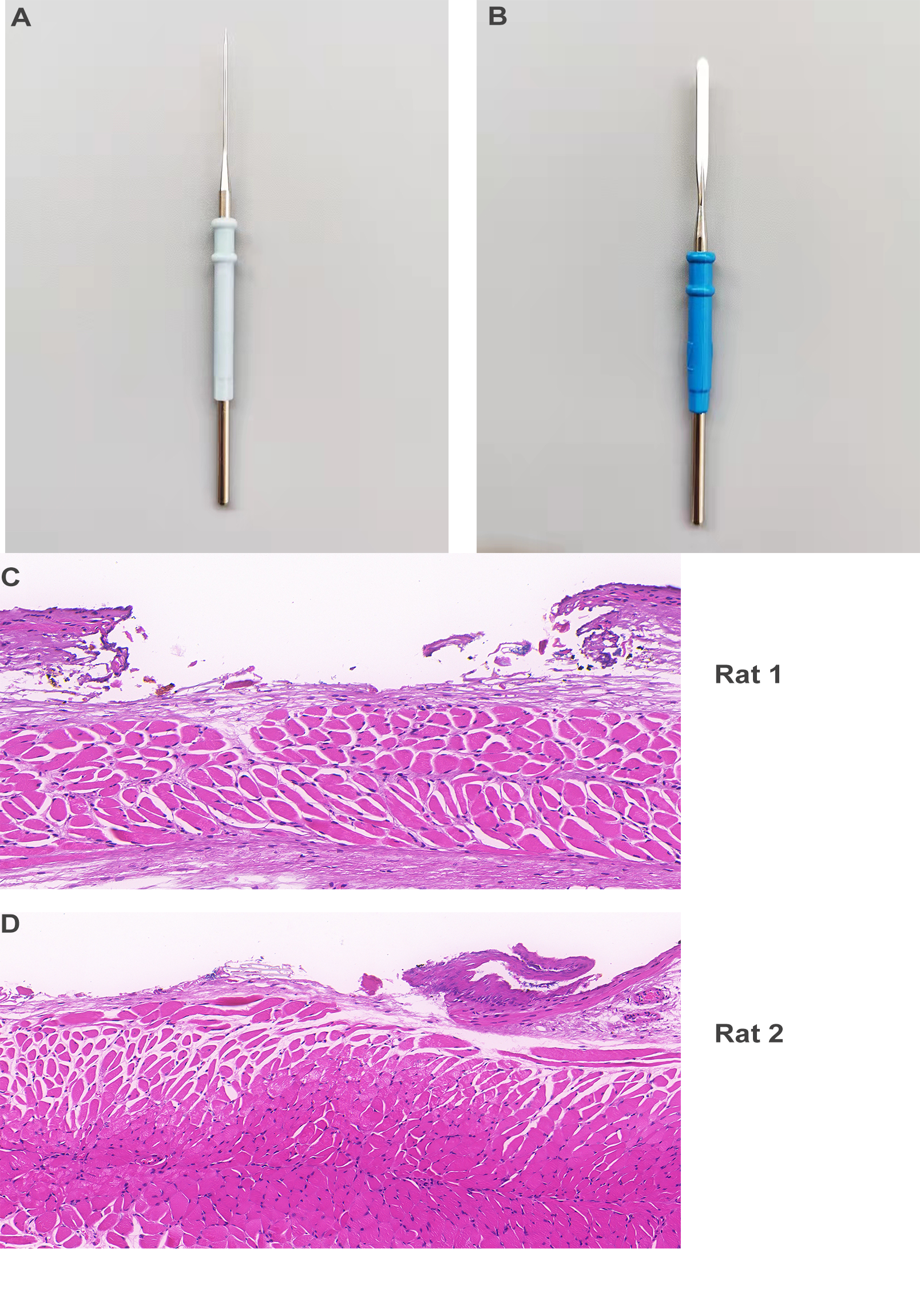

Supplement: Supplementary file 1 — Supplementary Figure 1. [file 41598_2023_38575_MOESM1_ESM.tif]

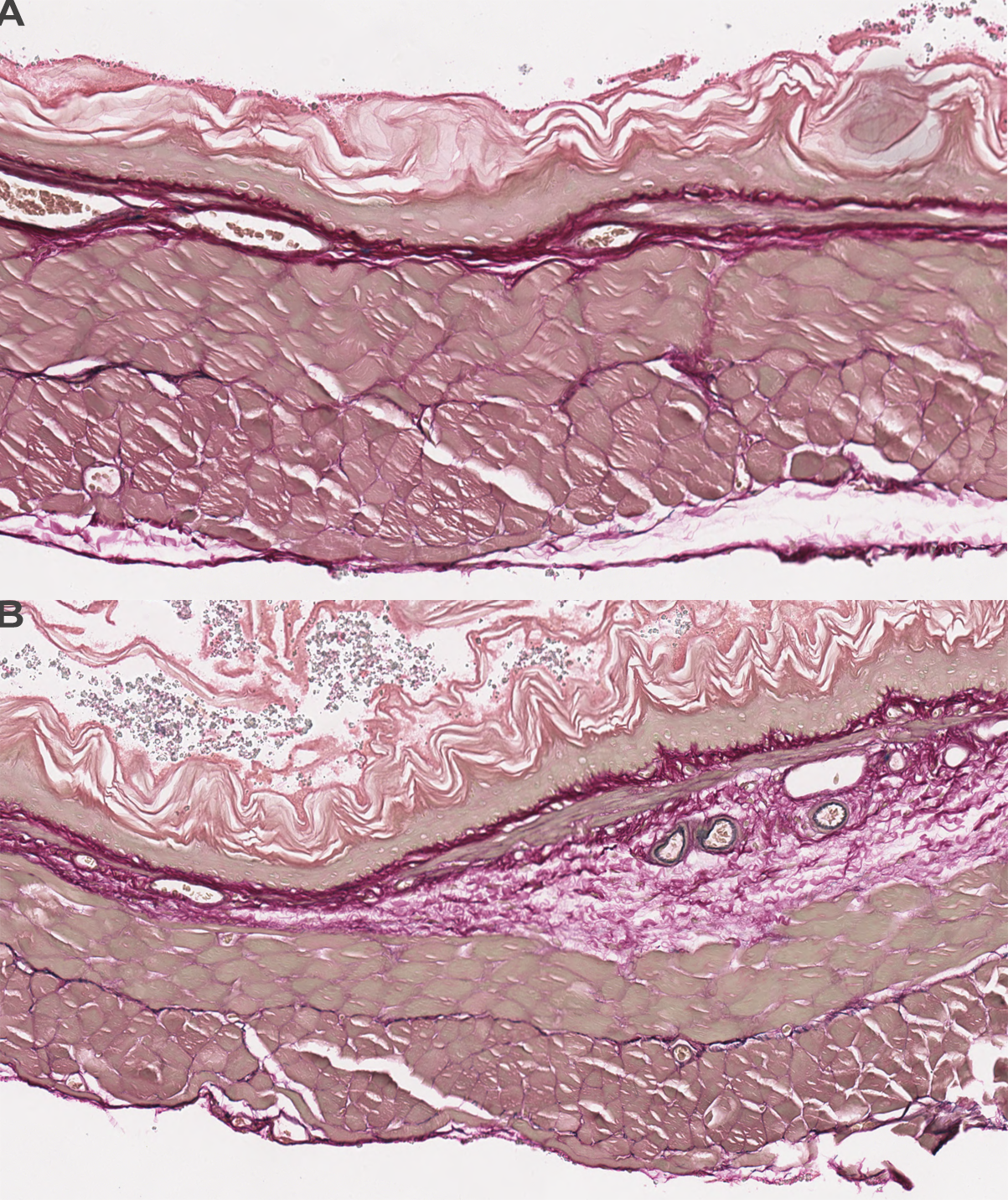

Supplement: Supplementary file 2 — Supplementary Figure 2. [file 41598_2023_38575_MOESM2_ESM.tif]

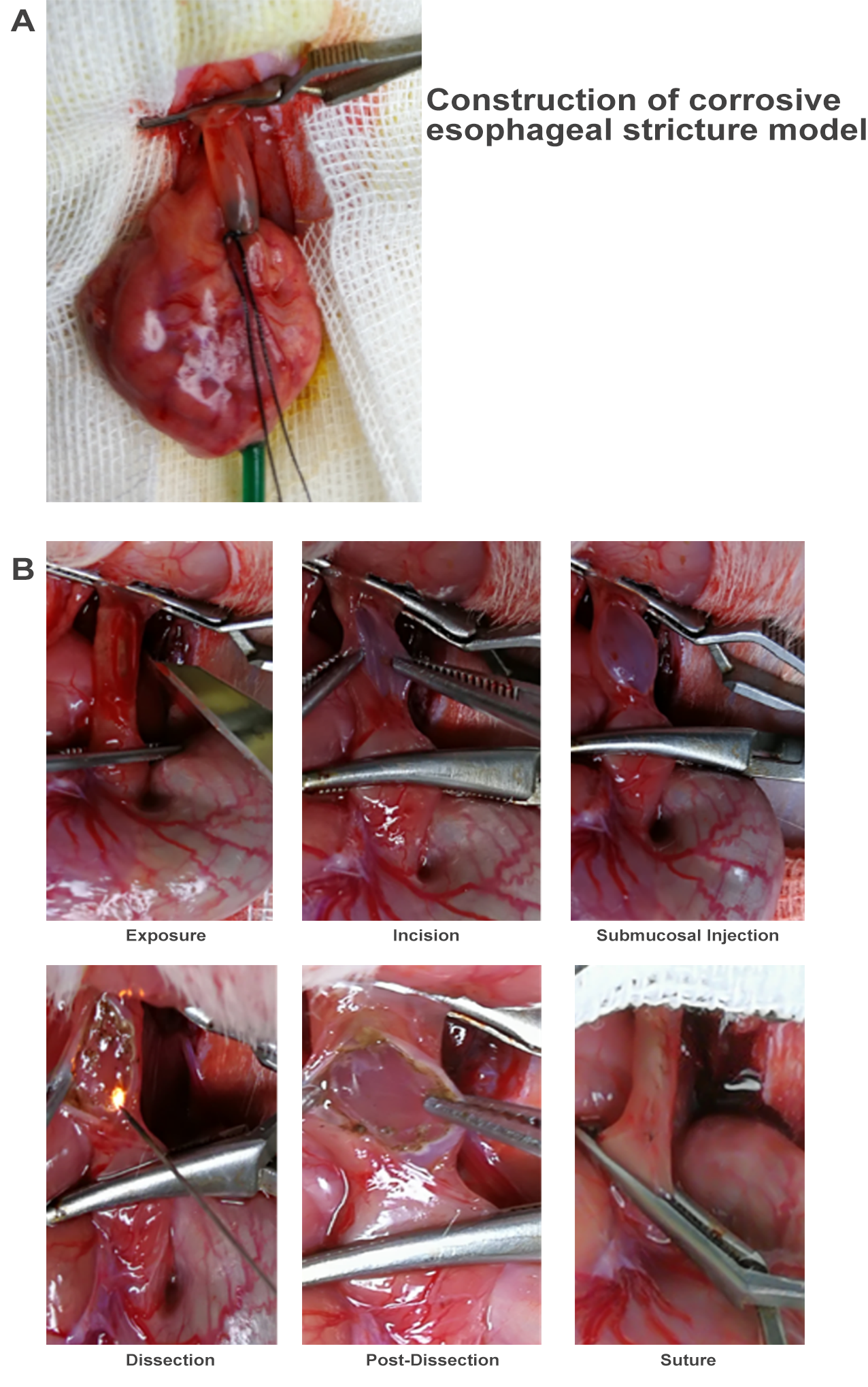

Supplement: Supplementary file 3 — Supplementary Figure 3. [file 41598_2023_38575_MOESM3_ESM.tif]
