# Supplementary material for: Benign esophageal stricture model construction and mechanism exploration
Source: Sci Rep. 2023 Jul 20;13:11769. doi: 10.1038/s41598-023-38575-y (PMC10359281; doi:10.1038/s41598-023-38575-y)
Supplement: Supplementary file 4 — Supplementary Legends. [file 41598_2023_38575_MOESM4_ESM.docx]

**Supplement Figure Legends:**

**Supplement Figure 1 Constructing rat model of esophageal stricture.** A. The sharp electric knife. B. The flat electric knife. C-D. The mucosal layer of the esophagus was removed in the rat model, which is consistent with ESD operation.

**Supplement Figure 2 The Victoria blue staining of rats in the model group. A.** The Victoria blue staining of periscar tissue for rats in model group. **B.** The Victoria blue staining of scar tissue for rats in model group.

**Supplement Figure 3 Constructing rat model of esophageal stricture with corrosion or surgery. A.** Constructing rat model of esophageal stricture with corrosion. **B.** Constructing rat model of esophageal stricture with surgery.
